# Nuclear Translocation Triggered at the Onset of Hearing in Cochlear Inner Hair Cells of Rats and Mice

**DOI:** 10.1007/s10162-023-00894-2

**Published:** 2023-03-17

**Authors:** Megana R. Iyer, Radha Kalluri

**Affiliations:** USC Caruso Department of Otolaryngology-Head and Neck Surgery, Zilkha Neurogenetic Institute, University of Southern California, Los Angeles, CA USA

**Keywords:** Nuclear translocation, Cochlea, Hair cells, Onset of hearing

## Abstract

**Purpose:**

Nuclear position is precisely orchestrated during cell division, migration, and maturation of cells and tissues. Here we report a previously unrecognized, programmed movement of the nucleus in rat and mouse cochlear inner hair cells (IHCs) coinciding with the functional maturation of inner hair cells around the onset of hearing.

**Methods:**

We measured hair cell length and nuclear position from confocal scans of immunofluorescence-labeled hair cells from whole-mount cochlear preparations throughout post-natal development.

**Results:**

In early post-natal days, the IHC experiences a period of sustained growth, during which the nucleus sits at the very basal pole of the cell, far from the apically located mechano-transducing stereocilia, but close to where synapses with primary afferent and efferent neurons are forming. After IHCs reach their final length, the nucleus moves to occupy a new position half-way along the length of the cell. Nuclear translocation begins in the middle turn, completes throughout the cochlea within 2–3 days, and coincides with the emergence of endolymphatic potential, the acquisition of big-conductance potassium channels (BK), and the onset of acoustic hearing. IHCs cultured in-vitro without endolymphatic potential (EP) do not grow, do not express BK, and do not experience nuclear movement. IHCs cultured in high K+ solutions (to simulate EP) grow but do not experience nuclear movement or acquire BK channels.

**Conclusion:**

Nuclear migration at the onset of hearing is a key step in the morphological maturation of IHCs. Whether this plays a role in functional maturation remains to be explored.

## Introduction

The position of a cell’s nucleus is important for supporting various cellular functions. The precision with which the nucleus is maneuvered in polarizing, migrating and mitotic cells highlights the importance of nuclear position at different points in a cell’s development. For example, in developing neuroepithelia, nuclei move between the apical and basal poles of the cell during each phase of the cell division cycle in a process called interkinetic nuclear migration [1]. The apical position during division ensures that the two daughter cells each receive a nucleus and properly integrate into the rest of the tissue [2]. Disruption of this process leads to unbalanced cell fate decisions, aberrant differentiation [3], and loss of epithelial structure [4].

Emerging evidence from several systems suggests that nuclear position is also important in differentiated post-mitotic cells. Hearing, vision, and muscular pathologies have all been noted from misplaced nuclei (reviewed in [5]). In the outer hair cells of the mammalian auditory system, mutations that disrupt the links between the nuclear envelope and cytoskeleton cause nuclear misplacement leading to cell degeneration and deafness [6]. Similarly, in cone photoreceptors, the nucleus moves along the basal–apical axis of the cell during the final phases of post-natal development [7]. Disruption of the **Li**nkers of **N**ucleoskeleton to **C**ytoskeleton (LINC) complex prevents this movement, resulting in reduced synaptic efficiency and retinal dysfunction [8].

The reason why nuclear position matters for these cells remains unclear. One possibility is that the influence of extrinsic signals on nuclear transcription is regulated by nuclear position. Alternatively, genetic programs responding to developmental cues may direct construction of specialized cellular structures such as the ribbon synapse and mechano-transduction machinery. Accurate construction of these structures may require the careful positioning of the nucleus within the cell. For instance, in multi-nucleated muscle cells, nuclei are normally positioned at the peripheries of the cell and close to synapses. The proximity to synapses appears to promote appropriate synaptogenesis as nuclear mispositioning results in improper synaptic development and muscle dysfunction [5].

Here, we describe a post-mitotic nuclear migration in the inner hair cells of the mammalian cochlea. This movement is remarkably abrupt and coincides with the onset of hearing, when inner hair cells’ physiology transforms from spiking (i.e., producing action potentials) to non-spiking (reviewed in [9]). In contrast, nuclear position does not change in outer hair cells, whose nuclei remain polarized to the base of the hair cell throughout post-natal development. Using immuno-histochemistry, we characterized the timeline of nuclear migration in inner hair cells and compare this to previously reported changes in inner hair cell morphology and physiology. We propose and test the idea that nuclear migration and the functional maturation of inner hair cells are linked to the dramatic increase in endolymphatic potential around the onset of hearing. Our results suggest that the signaling cascades that drive the final phases of inner hair cell maturation and nuclear movement are not simply recapitulated by a simple manipulation that tonically depolarizes inner hair cells.

## Methods

All procedures were approved by the animal care and use committee at the University of Southern California. Chemicals were obtained from Sigma-Aldrich (St. Louis, MO), unless otherwise specified.

### Animals

Data in this paper were collected from 30 Long-Evans rats (Charles River Laboratories) ranging in age from postnatal day (P)3–69 and 7 wildtype CD1 mice (Jackson Labs) ranging in age from P10-P20. All animals were handled and housed in accordance with National Institutes of Health *Guide for the Care and Use of Laboratory Animals.* All animal procedures were approved by the University of Southern California Institutional Animal Care and Use Committee.

### Cochlear Dissection and Immunostaining

Cochlear samples were prepared from rats ranging in age from P3 through P69. Otic capsules were dissected in chilled and oxygenated Leibovitz-15 (L-15) medium supplemented with 10 mM HEPES (pH 7.4, ~315 mmol/kg). Samples were fixed in 4% PFA for 60–90 min at room temperature. At older ages (P18 and above), samples were decalcified in 10% EDTA for 2–5 days before fine dissection. Between 3 and 7 cochleae contributed to the data set at most ages.

Fixed cochleae were transferred to phosphate-buffered saline (1 × PBS, pH 7.4). Fine dissection began by removing the bony covering of the otic capsule to reveal three turns of the cochlea. Sharp scissors were used to separate the apical, middle, and basal turns. The stria vascularis, excess bone, and other overlaying membranous layers including the tectorial membrane were removed using fine forceps. Turns were incubated in a blocking buffer containing 16% normal goat serum, 0.03% Triton X-100, 120 mM phosphate buffer, and 450 mM NaCl for 1 h at room temperature. After three rounds of wash in 1 × PBS for 5 min each, preparations were incubated in dilutions of primary antibodies at room temperature for 16–20 h or at 4 °C for 72 h. Secondary antibodies were then added and incubated for one hour at room temperature. Following the primary and secondary incubations, we washed the samples for three 10-min rounds in 1 × PBS.

The following primary antibodies were used to label the following structures: (1) anti-CtBP2 mouse monoclonal IgG (Becton Dickinson Company) diluted 1:250 to label the synaptic ribbon-specific protein RIBEYE (validated in [10]), (2) anti-myosin-VI-rabbit polyclonal (Proteus Bioscience Inc.) diluted 1:500 to label the cytoplasm of inner hair cells (validated in [10]), (3) anti-KCNMA (Alomone Labs, diluted 1:500 and validated in [11]) and in select samples, (4) anti-beta III tubulin mouse IgG2a diluted 1:250 (TUJ1; Covance) to label cochlear neurons (validated in [10]). The following secondary antibodies acquired from Life Sciences Inc. were used and diluted 1:250: Alexa Fluor 488 anti-mouse and Alexa Fluor 594 anti-rabbit. Samples at different ages were typically processed on different days when animals of the correct age became available. Samples from several experiment days were pooled to provide the data at any particular age.

A subset of measurements was made from cochlear scans collected as a part of another project. The goal of those experiments was to record from spiral ganglion neurons (see [12] for details). As such, the samples were not immediately fixed and the cochleae were first exposed to an enzymatic cocktail comprised of trypsin and collagenase in L-15. Hair-cell morphology was not noticeably different between the age matched samples collected for the present purpose and those that were reanalyzed from the previous data set. However, wherever possible, these data are distinguished by a different symbol.

### Organotypic Culture of Organ of Corti

Organs of Corti from animals aged P8 subjected to long-term culture for 4–5 days. Samples were dissected and cultured on membrane filters (SPI) in culture media (MEM, Glutamax Supplement, Fisher Scientific), supplemented with 10 mM HEPES. A subset of samples was cultured in culture media supplemented with 20 mM KCl solution for 4 days following 1 day in standard culture media. Samples were then fixed for 10 min in 4% PFA, and labeled for Hoechst, Myosin VI, CTBP2, and big-potassium labeling antibody Anti-KCNMA1 (Alomone Labs).

### Imaging

Fixed and labeled samples were mounted under a glass coverslip and onto glass slides with the fade-protectant medium Vecta-shield (Vector Laboratories). Hardened nail polish dots were placed under the coverslip to prevent it from crushing the cochlear sections. We generated a series of z-stack images of immunofluorescent signals using one of the following two confocal microscopes (1) Olympus FV1000 laser scanning confocal microscope and (2) Zeiss LSM 800 with a 40–63×, 1.42 numerical aperture, and an oil immersion objective. To visualize the entire inner hair cell, z-stack images were taken from the cuticular plate to the very basal pole of the cell. Each channel was scanned in sequence with the same optical thickness. Laser power, gain, and offset settings for each channel were set to optimize the dynamic range of the fluorescent signal in each sample (between 0 and 4095).

### Analysis

Analysis was done on two sets of data: the first set of images were taken from confocal scans of samples that were fixed after electrophysiological recordings [12], while the second were fixed immediately after euthanasia.

Both length calculations and the analysis of nuclear position in hair cells were done offline using Icy (http://icy.bioimageanalysis.org/). Images included in this paper were processed using Imaris software to produce 3D projections of samples.

**Fig. 1:**
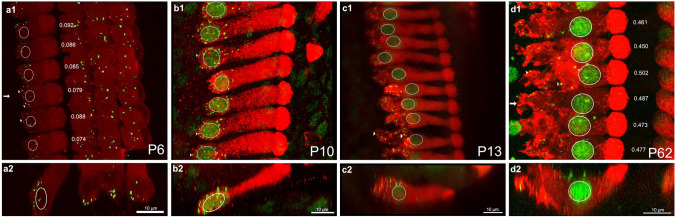
A dramatic change in nuclear position and hair cell morphology occurs around the onset of hearing. **a1–d1** Confocal images of inner hair cells in the middle cochlear turn at P6, P10, P13, and P62 in the XY plane. Inner hair cells were labeled with Myosin VI (red) and nuclei and synaptic ribbons (arrowheads) were labeled with CTBP2 (green). Normalized nuclear position measurements (defined in the “Methods” section) are shown next to each inner hair cell. **a2**–**d2** Same samples as above shown as a cross-section in the XZ plane to view the length of the entire inner hair cell. **a1**, **b1** Before the onset of hearing, nuclei appear to be located at the base of IHCs and synaptic ribbons are clustered around the nucleus. **c1** By P13 in the middle turn, variance in nuclear position is apparent. Ribbons have dispersed throughout the basal pole. **d1** By P62, the nucleus has reached its final position in the middle of the cell. A full migration seems to have occurred by P62. Outer hair cells, though present and labeled, were cropped out of these scans

Measurements were taken in the XY and XZ planes. The 3-D region of interest tool was used to place a point at the end of the cuticular plate, at the top end of the nucleus closest to the basal pole, and at the basal-most region of the inner hair cell (see arrows in Fig. 1, a2). For hair cells that appeared bent, a fourth point was placed at the base of the nucleus, closest to the cuticular plate, to accurately capture the full length of the cell.

Lengths were calculated using all three dimensions of each point. L1 was defined as the supranuclear length, or the length between the cuticular pole and the top of the nucleus, and L2, the subnuclear length, was defined as the length between the top of the nucleus and the basal pole. Nuclear position was calculated by dividing L2 by the total length of the hair cell (L1 + L2); here, we normalized by cell length to account for differences in length across the cochlea and with maturation. Normalizing by length was also necessary to account for fixation or mounting artifacts that may have caused stretching of the cells.

### Statistical Analysis

Welch’s ANOVA with a post-hoc Games Howell test was used to compare the following parameters: nuclear position in pre-hearing IHCs and post-hearing IHCs; and nuclear position as a function of cochlear turn at a critical period. The level of significance for this study was *p* < 0.05.

## Results

We imaged inner and outer hair cells (IHCs and OHCs, respectively) from the apical, middle and basal turns of cochleae in rats ranging in age from P3 to P69. Examples of labeled inner hair cells and nuclei from the middle cochlear turn are shown in the XY and XZ dimensions at P6, P10, P13, and P62 (Fig. 1a–d). Nuclei and synaptic ribbons were fluorescently labeled using an antibody against c-terminal binding protein 2 (CtBP2, green). We visualized the cytoplasm and boundaries of the hair cells by fluorescently labeling with antibodies against the motor protein, Myosin VI (red).

The positions of inner hair cells’ nuclei change dramatically during post-natal maturation. During the first two post-natal weeks (e.g., P6 and P10, Fig. 1a, b), nuclei are located at the very base of IHCs, leaving little Myosin VI labeled area under the nucleus. Synaptic ribbons are found tightly clustered near the nucleus at the basal pole of the cell (arrowheads). By contrast, in mature inner hair cells, nuclei are found near the middle of the hair cell (Fig. 1d) with a substantial sub-nuclear region (Myosin VI enriched). Ribbons (arrowheads) are found at the basal periphery of the sub-nuclear region and well below the mid-line position of the nucleus. At P13, nuclear position is highly variable and ranges between the basal pole and middle of hair cells (Fig. 1c).

**Fig. 2:**
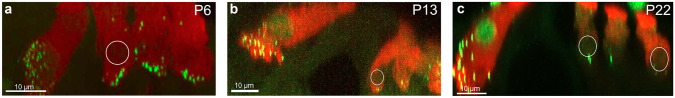
Outer hair cell nuclei remain basal bound throughout development. Outer hair cells in the middle turn are outlined at P6 (**a**), P13 (**b**), and P62 (**c**). Unlike inner hair cells, nuclei in OHCs are positioned at the basal pole of the cell in all samples

The change in the nuclear position of inner hair cells is contrasted by the relative age invariance of nuclear position in outer hair cells. Nuclei in outer hair cells remain located at the bottom of the cell throughout early post-natal development (Fig. 2a) and throughout maturation (Fig. 2b, c).

**Fig. 3:**
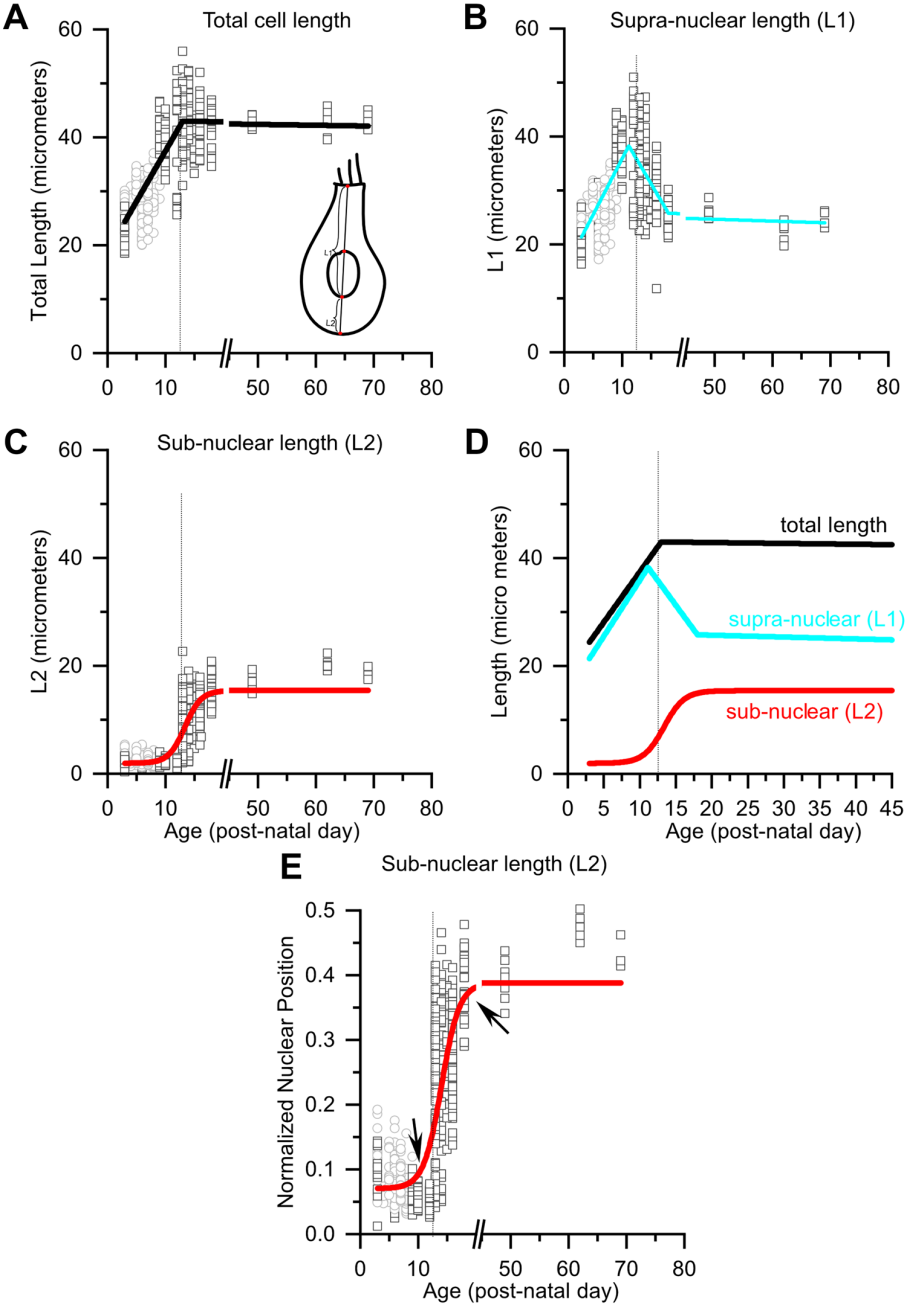
Quantification of nuclear position reveals a period of progressive cell growth followed by an abrupt nuclear translocation event at the onset of hearing. **a** Total IHC length as a function of post-natal age ranging from P3 to P69 pooled across all turns. Open squares are length measurements from individual hair cells from samples in which cochleae were fixed immediately after euthanasia for immunohistochemistry. Open circles indicate samples in which sample cochleae were fixed after electrophysiological recordings [12]. Cell length grows progressively until it reaches a maximum around P13. **b** Supra-nuclear length (*L*1) increases progressively as a function of age until the onset of hearing when it suddenly begins to decrease, eventually reaching a stable length. **c** Sub-nuclear length is relatively small until about P13 when it begins to increase, eventually reaching a stable length. **d** The trendlines describing total (black), supra-nuclear (cyan), and sub-nuclear lengths (red) are drawn on the same axis to compare the timelines for age dependent changes in lengths. The coincidence between the decrease in supra-nuclear length with the increase in sub-nuclear length indicates that the nucleus is moving. **e** Relative Normalized nuclear position, which characterizes nuclear position while accounting for length changes (*L*_2_ / (*L*_1_ + *L*_2_)), increases suddenly around P13 (first arrow) and stabilizes by P18 (second arrow). The inset schematic in **a** shows how different lengths were measured. The dashed line in all plots is drawn at P13. Trend lines were drawn either by piecewise linear fitting (**a**, **b**) or by a Boltzmann fit (**c**–**e**)

To characterize the timeline over which nuclear position changes in inner hair cells, we quantified nuclear position relative to total hair cell length as a function of post-natal age (Fig. 3). To do this, we measured a series of high-resolution confocal scans through the inner hair cells (e.g., the images in Fig. 1). Next, we bisected each cell using an oblique slice through its long axis (this was typically very close to a plane in the XZ dimension). Then we measured the length of the subnuclear and supranuclear regions of each cell (Fig. 3a, inset). The subnuclear length, ${L}_{2}$, was measured from the bottom of the nucleus (the point closest to the basal pole) to the basal-most point of the hair cell. Supranuclear length, ${L}_{1}$, was measured piecewise as the sum of two lengths: the nuclear length plus the length from top of the nucleus to the cuticular plate. The sum of ${L}_{1}$ and ${L}_{2}$ was taken as the total cell length. Note that the piece-wise measurement was effective at capturing cell length even in samples where the hair cell appeared to bend (e.g., Fig. 1d2). In addition to raw lengths, we computed nuclear position relative to the total length of the cell: $N=\frac{{L}_{1}}{{L}_{1}+{L}_{2}}.$ The relative position considers sample to sample variations in cell length that might arise due to maturation or methodological variability.

### Nuclear Position Changes Because Nuclei Move and not Because Hair Cells Grow

Total hair cell length increases progressively as a function of age during the first two post-natal weeks, at which point it reaches a maximum and length becomes relatively age-invariant (Fig. 3a). A maturational increase in cell length is consistent with previous electrophysiological reports showing that hair cell capacitance increases with post-natal age [13].

**Fig. 4:**
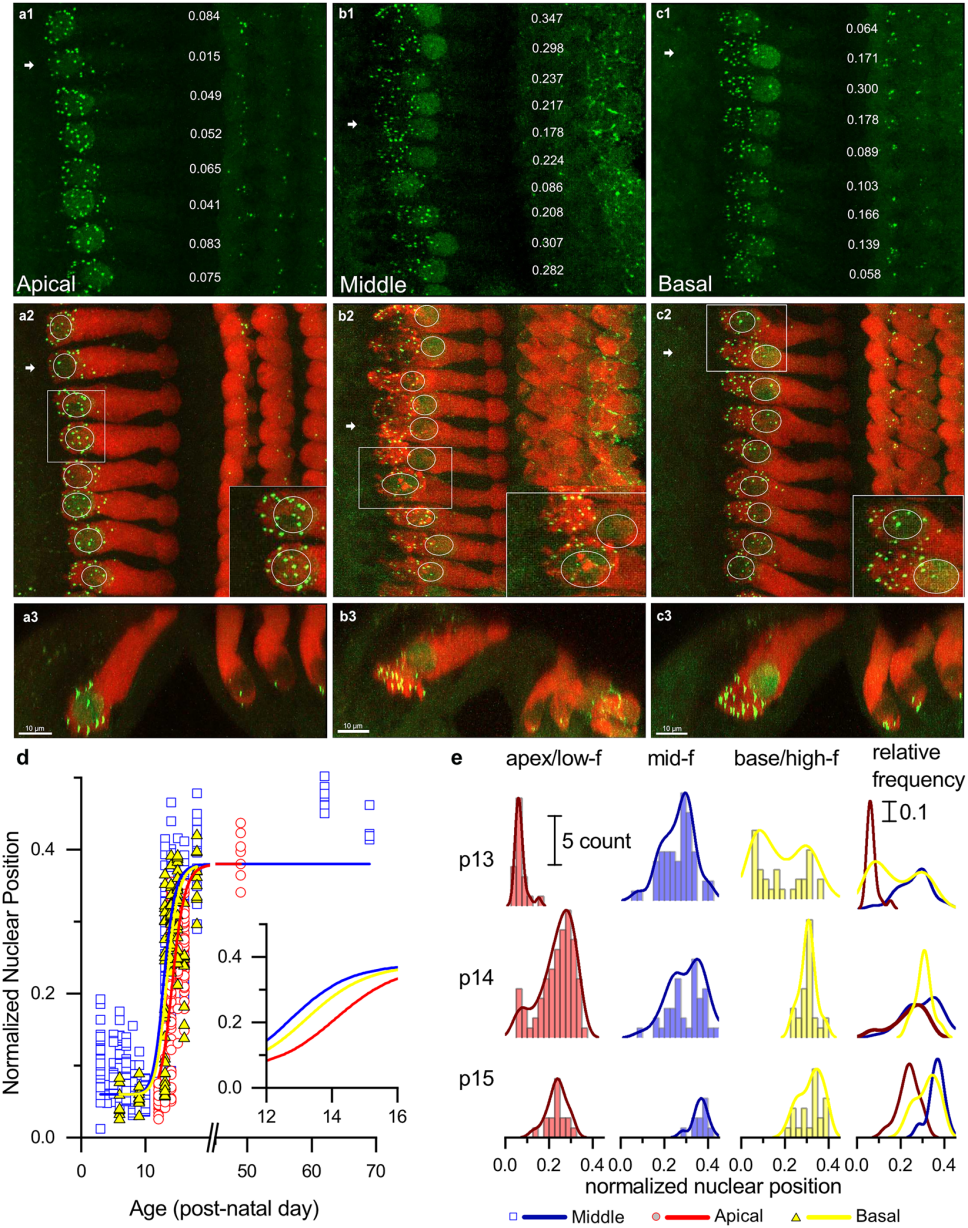
Nuclear translocation happens first in the middle and basal turns and then in the apical turn. **a1**–**c1** CTBP2 labeled nuclei and ribbons in apical, middle, and basal turns from the same P13 sample. **a2**–**c2** Merged images of inner hair cells labeled with Myosin VI (red) and CTBP2 (green) in the XY plane. In the apical turn, ribbons are clustered around the nucleus, which is bound to the base of the hair cell. Nuclear position is variable in the middle turn, and ribbons are dispersed across the basal pole in both the middle and basal turns. **a3**–**c3** Images of all three turns at the XZ plane. The images are shown through the hair cell indicated by the white arrows in A2-C2.in the XZ plane. Here, outer hair cells are visible and OHC nuclei were located at the base of cells in all three turns. At P13, most nuclei in the apical turn inner hair cells are found at the base of the cell. In contrast, nuclear position is more variable in the middle and basal turns. Note the instances of adjacent cells with similar lengths but different nuclear positions (insets). Variance in nuclear position across adjacent cells suggests that this sample has hair cells caught in the middle of transitioning between two distinct developmental states. **d** Normalized nuclear position as a function of age for all three cochlear turns from P3-P69. Nuclear migration in the middle turn (blue curve) begins earliest, followed by migration in the basal and apical turns (yellow and red, respectively). Nuclear migration in the apical turn lags 1–2 days behind the middle turn. **e** Histograms showing relative normalized nuclear position in apical (red), middle (blue), and basal (yellow) turns at P13, P14, and P15. Smoothed distributions are overlaid on top of each histogram and compared on the same axis. Note that the distributions are largely bimodal with one mode at approximately 0.07 and another at 0.4. The heights of the two modes indicate the relative developmental state of the groups of hair cells, with apical hair cells lagging behind the basal turn and middle turns

As we noted in Fig. 1, nuclear position changes dramatically before and after the onset of hearing. Given that cell length changes during maturation, changes in the relative position of the nucleus from the basal pole to the middle of the cell (e.g., Fig. 1) could result from either an outgrowth of the sub-nuclear region and/or a migration of the nucleus. To determine which of these scenarios is at play, we examined age-dependent changes in the sub-nuclear and supra-nuclear lengths of the inner hair cells (Fig. 3b, c). Much like total cell length, supra-nuclear length, which is the length from the apical end of the nucleus to the cuticular plate (see Fig. 3a, inset, L1), grows progressively with age, reaching a maximum around P14 (Fig. 3b). During this period, sub-nuclear lengths, which are measured from the base of the nucleus to the basal-most end of the cell (see Fig. 3a, inset, L2), are small and do not change (Fig. 3c). In other words, the nucleus is basal bound while the cell grows. Then, around P14, an abrupt change occurs, and sub-nuclear lengths suddenly begin to increase, while supra-nuclear lengths decrease. Note that by this time the cell length has stopped changing (compare the time course for total, sub-nuclear, and supra-nuclear length changes in Fig. 3d). The simultaneous increase in sub-nuclear length coupled with a decrease in supra-nuclear lengths, while total length stays constant, indicates that the nucleus is moving from the bottom to the middle of the cell. That nuclei are migrating — as opposed to the basal pole expanding below the nucleus is also evident when comparing adjacent cells of similar lengths in the middle turn, which have nuclei at very different positions (e.g., Fig. 1c.1. See also Fig. 4b, c). This variance in nuclear position indicates that these images caught adjacent cells at different stages of nuclear translocation.

These results indicate that after a period of progressive cell growth, the inner hair cell suddenly stops growing and the nucleus migrates from the basal pole to the middle of the hair cell. We illustrate the time course of nuclear migration by plotting the normalized nuclear position (*N*) versus post-natal age in Fig. 3e. This figure clearly indicates that the onset of nuclear migration begins rapidly around P12 and stabilizes by P16 (see arrows, Fig. 3e). The start of this nuclear repositioning event coincides with the onset of acoustic hearing in rats, when the ear canal opens and hair cells transition from spiking to non-spiking cells (reviewed in [9]), note the light vertical line in all panels of Fig. 3, which corresponds to the onset of acoustic hearing in rats (P12 in rats).

### Nuclear Migration Occurs First in the Middle Cochlear Turn

Next, we examined the time course of nuclear translocation across the cochlear length in rat. To do this we sorted the data shown in Fig. 3e according to one of three cochlear turns: the low-frequency encoding cochlear apical turn, the mid-frequency encoding cochlear middle turn, and high-frequency encoding cochlear basal turn. Before P12, nuclei appeared to be positioned at the bottom/basal pole of the inner hair cell in all three turns. By P13, IHCs in the middle turn showed signs of a migration event (compare the nuclear positions from the same cochlea in Fig. 4a–c, see also the insets). Nuclei in the apical turn, however, are almost exclusively found at the basal pole of the inner hair cell (Fig. 4a). Note the striking variability in nuclear positions in the three cochlear turns.

When compared to the observed timeline in the middle turn, the onset of nuclear translocation is delayed in apical and basal turn cochleae, as illustrated in Fig. 4d and e. In 4d, the relative nuclear position across age is shown with the data now color coded to indicate cochlear turn. The Boltzmann curves through the nuclear position data are shifted to the right for the basal and apical turns when compared to the middle turn.

To further quantify differences across cochlear turn, Fig. 4e shows the distribution histograms of nuclear position at P13, P14, and P15. The smoothed distribution curves that are overlaid on the histograms are largely bimodal, with one mode at the bottom of the hair cell (nuclear positions around 0.07) and the second mode near the middle of hair cells (nuclear positions around 0.4). The relative height of each mode indicates the proportion of nuclei that are in either the immature position at the bottom of the hair cell or the mature position in the middle of the hair cell. In the apical turn at P13, most of the inner hair cells’ nuclei are in first mode at the immature position. In the middle turn, many nuclei are already in the mature second mode. The basal turn is more advanced than the apical turn in having some nuclei that have moved to the mature position but perhaps not as advanced as the middle turn because many nuclei are still in the immature position.

By P14 and P15 nuclear position of both middle and apical turn hair cells has progressed, with most nuclei in the apical turn now occupying the second mode, although nuclear position is still significantly different between middle and apical turns (*F*(1, 58) = 26, *p* < 0.0001, Welch’s *t*-test). In summary, nuclear migration begins in the middle turn and is delayed in the basal and apical turns.

**Fig. 5:**
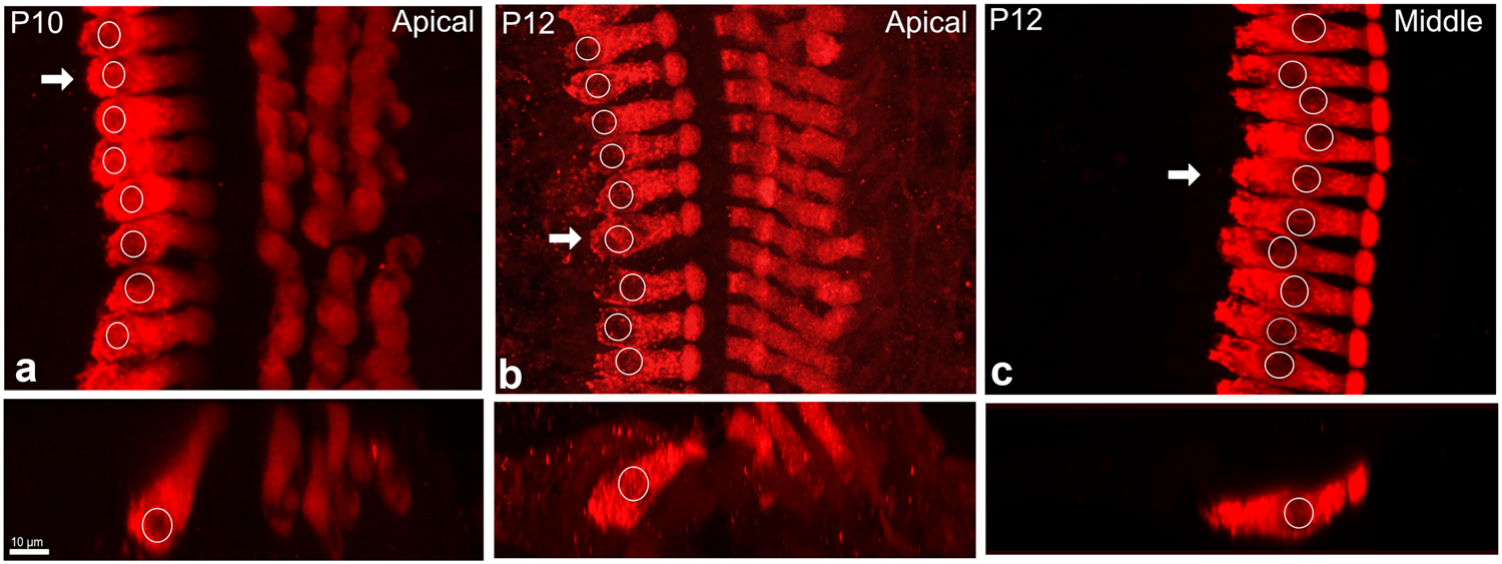
Nuclear migration occurs earlier in mouse than rat. **a** Nuclei in the apical turn in wildtype CD1 mice at P10 are located at the base of the inner hair cell. **b**, **c** P12 inner hair cells from the apical and middle turns of the same cochlea. Nuclei have begun to move in the apical turn, and have reached their final position by the middle turn

To validate our results in the developing rat, cochleae from wildtype CD1 mice (3 at P10; 4 at P12; 3 at P13) were extracted and IHC nuclear position was analyzed. Nuclear position migrates from the basal pole to the middle of the inner hair cell during post-natal development in mouse as it does in rat. At P10 in the apical turn, mouse inner hair cells have nuclei positioned close to the basal pole of the cell (Fig. 5a). By P12, a few nuclei in the apical turn start to migrate (Fig. 5b). However, in the middle turn of the same P12 cochlea, most nuclei are already positioned in the middle of the inner hair cell, near their final mature position (Fig. 5c). Based on these data, nuclear migration is happening in both rat and mouse, perhaps a little earlier in mouse consistent with their earlier onset of hearing (discussed in [14]). Thus, nuclear migration appears to be a general feature of post-natal inner hair cell development.

### Tonic Depolarization in Organotypic Culture Stimulates Hair Cell Growth but not Nuclear Migration

**Fig. 6:**
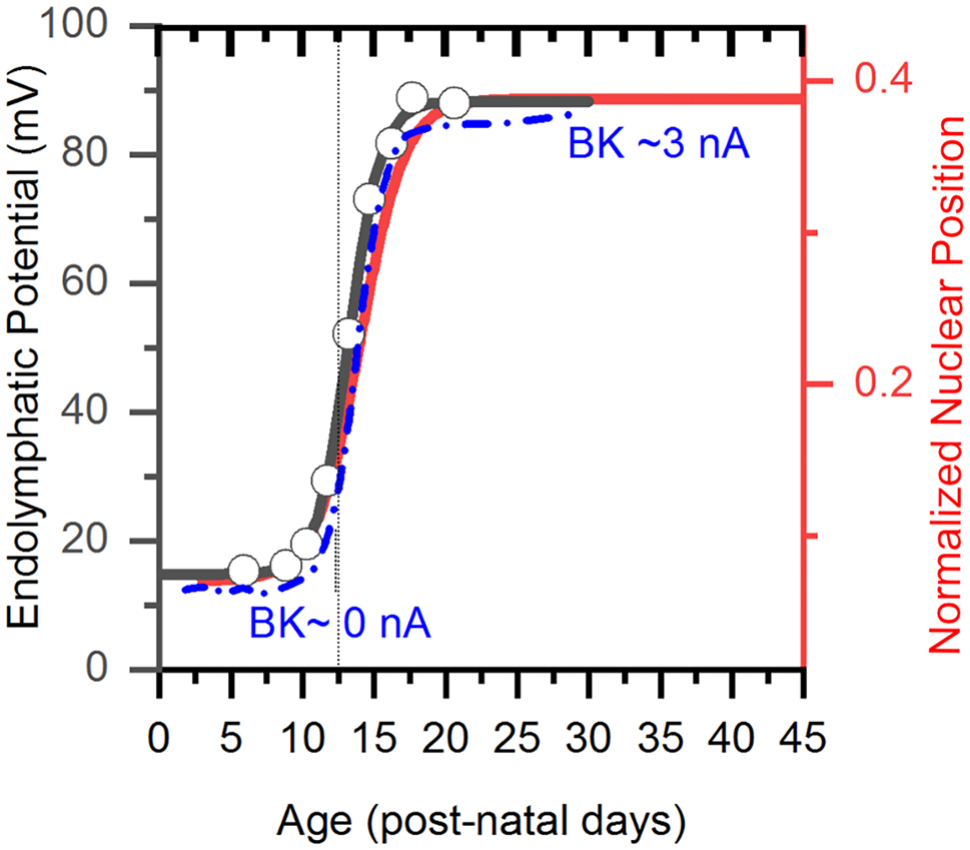
Inner hair cells acquire BK channels in coincidence with EP, nuclear migration, and the onset of hearing.** f** The emergence of EP (Black curve and y-axis on left [18]) in rat IHCs coincides with the onset of nuclear translocation (red curve, y-axis to the right); both events are abrupt and occur just at the onset of hearing. The age axis for EP data was adjusted from the originally reported gestational age to post-natal day. The emergence of BK channel currents (blue curve, reproduced from [17]) follows the same time course as nuclear migration and the onset of EP reported by Bosher and Warren. Note that the BK current data are from mouse, whereas EP and nuclear position data are from rat

The onset of hearing marks a period when the inner hair cell undergoes substantial biophysical changes — including the emergence of KCNQ-type K+ channels, disappearance of small potassium (SK) channels [15] and the retreat of efferents from IHCs [16]. Amongst the most striking ion channel changes is the acquisition of big-conductance calcium-gated potassium channels (BK) [17]. Notably, the time and rapidity with which the nucleus migrates and when IHCs acquire BK channels coincides with the rapid emergence of endolymphatic potential (Fig. 6; EP data from [18]; BK currents, blue curve adapted from [17]).

In the following in vitro experiments, we tested the hypothesis that the sudden tonic depolarization that the onset of EP induces in inner hair cells triggers the morphological and electrophysiological maturation of inner hair cells. That EP plays a part in maintaining the mature electrophysiological phenotype of inner hair cells is supported by the observation that a tonic depolarization of inner hair cells is required to maintain BK channel expression in mature hair cells [13]. Here we test if a similar manipulation that causes tonic depolarization can induce the expression of BK channels in immature hair cells. We measured the following three features (that are easily measured by immunohistochemistry) as representative of hair cell maturation: (1) cell length, (2) nuclear position, and (3) expression of BK channels.

**Fig. 7:**
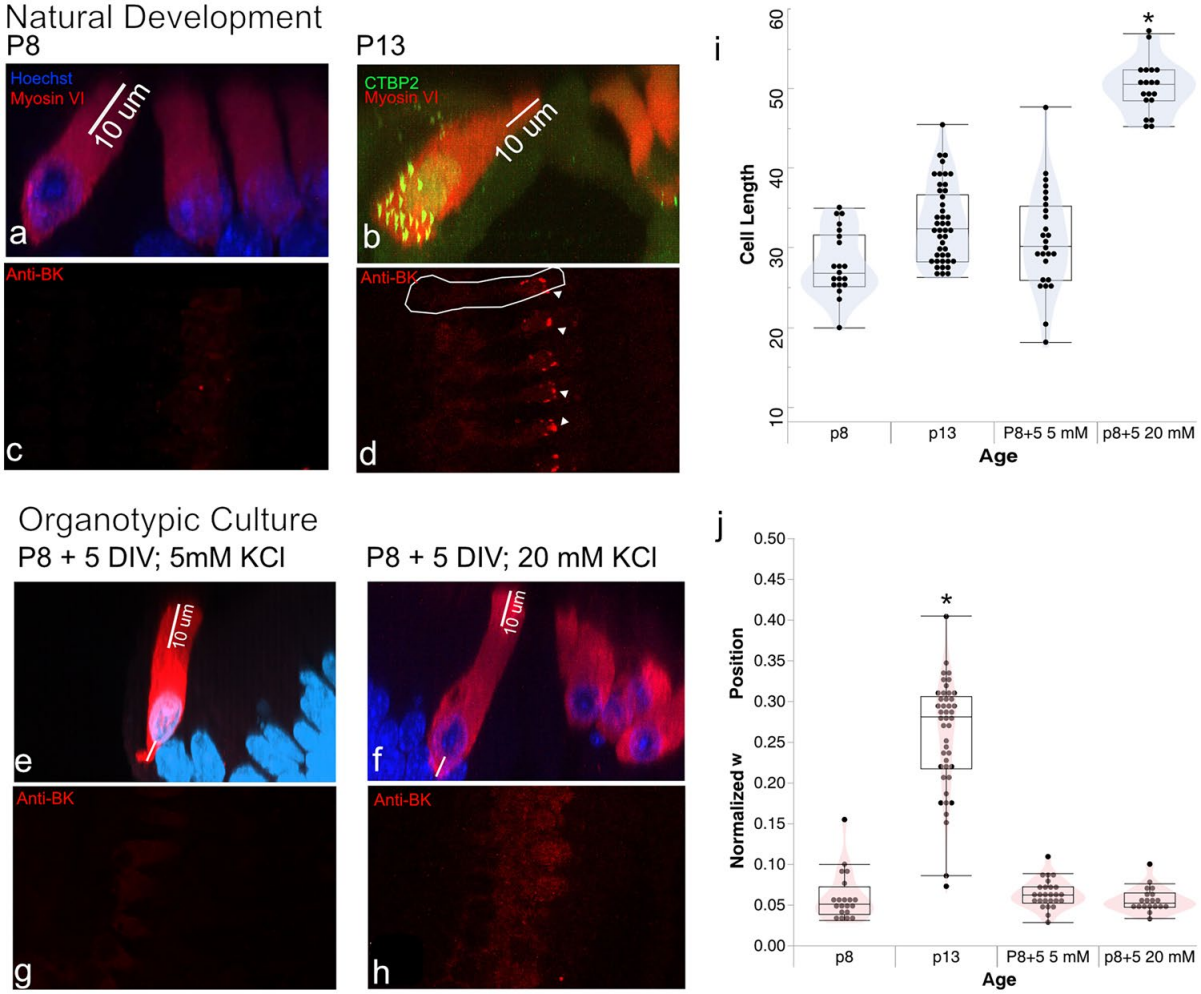
Tonic depolarization in organotypic culture stimulates hair cell growth but not nuclear migration** a** Inner hair cells from a P8 middle turn in rat appear immature in nuclear position and lengths. **b** Inner hair cells from a P13 middle turn have reached their final cell length.** c**, **d** P8 inner hair cells have not acquired BK channels, as noted by the absence of BK labeling at this age. At P13, red puncta (see triangles) signal the presence of BK channels at the apical end of the inner hair cell. The white outlines the hair cell. **e** Inner hair cells cultured for 5 days in culture media with 5 mM KCl.** f** Inner hair cells cultured for 5 days in culture media supplemented with 20 mM KCl. Cells grown in KCl were longer than those in 5 mM KCl, although BK channels were not visible in either condition (**g**, **h**).** i** Cell length was measured in samples from each condition; inner hair cells grown in 20 mM KCl were significantly longer than cells in all other conditions (*p* < 0.0001). **j** Inner hair cell nuclei extracted from rats at P13 have dislodged from the basal pole. In all other conditions, nuclei appear at the base of the inner hair cell

Here, we examined hair cell development in organotypic culture where hair cells would not experience the normal onset of endolymphatic potential. We cultured immature organs of Corti from P8 Long-Evans rats for up to 5 days. In natural development, between P8 and P13, inner hair cells experience cell growth, nuclear migration, and acquire BK channels (Fig. 7a, d). In addition to the 8 days of natural maturation, the culture period spans the net duration required to cover this period of development. For example, a sample plated at P8 and cultured for 5 DIV would have an effective age of P13. Three out of six organs of Corti were cultured for all 5 days in 5 mM KCl. The remaining samples were cultured in 5 mM KCl for 24 h and then transferred to culture medium supplemented with 20 mM KCl for up to 4 days. We estimate that the 20 mM KCl solution produces approximately 40 mV of depolarization to IHCs. Between 7 and 10 cells from the middle turn were analyzed in each cultured sample.

IHCs cultured in 5 mM KCl did not mature as they did during a similar time period in vivo. Their length did not change, their nuclei did not migrate (Fig. 7e) and their membranes did not acquire BK channels (Fig. 7g). By these three metrics, the maturation of cultured cells was effectively stalled. IHCs exposed to 20 mM KCl were longer than hair cells in all other conditions (Fig. 7f), indicating that tonic depolarization successfully stimulated cell growth. The average length of IHCs in 20 mM KCl was 50.18 microns (SD = 3.29), while those in 5 mM KCl measured 30.93 microns on average (SD = 6.25). IHCs cultured in 20 mM KCl were also larger both than those seen at P8 in normal development (27.95 microns, SD = 4.14) and at P13 in normal development (33.02 microns, SD = 4.89). Since lengthened cells are sometimes an indication of apoptosis, cells in two trials were labeled with an In-situ Cell Death Detection Kit (Roche). Hair cells did not label as indicating cell death (not shown), suggesting that lengthened IHCs had survived the period in culture. Results of a one-way ANOVA showed that growth conditions had a significant impact on cell length (*F*(3,105) = 82.47, *p* < 0.0001), with cells growing in 20 mM KCl being significantly longer than in all other conditions (Fig. 7i).

Although tonic depolarization appeared to stimulate the lengthening of cell, IHCs grown in 20 mM KCl did not acquire BK channels or undergo nuclear migration (Fig. 7h, j). The sub-nuclear length, L2, was not significantly different across the three conditions (P8; P8+5DIV 5 mM KCl; P8+5DIV 20 mM KCl) in which nuclear migration was not observed (*F*(2, 52) = 1.79, *p* = 0.18), indicating that nuclear migration did not occur. In summary, IHCs growing in organotypic culture do not automatically experience nuclear migration or acquire BK channels. Though inducing depolarization stimulates cell growth, this alone is not sufficient for triggering nuclear migration and maturational changes in ion channel composition. Thus, the signals that are present in vivo for driving the last functional steps of maturation remain missing in the culture conditions examined here.

## Discussion

### Nuclear Translocation Demarcates the Transition Between Two Distinct Periods of Post-natal Development in Inner Hair Cells

Here, we report that the nuclei of inner hair cells experience a remarkable sudden and timed migration from the bottom of the cell to the middle of the cell. This movement is notable because it coincides with the onset of hearing when these cells also experience a dramatic biophysical and functional transformation. Prior to the onset of hearing, inner hair cells produce regenerative action potentials independent of sensory input (e.g. [17]). This sensory-independent activity aids maturation by influencing cell growth, synaptogenesis, and the formation of upstream neuronal circuits [19–21]. Around the onset of hearing, inner hair cells cease to produce regenerative spikes and start responding with graded potentials that are useful for encoding the amplitude and timing of incoming sounds with high resolution. The correlation between the timing of nuclear migration and the functional transformation of the hair cells means that nuclear translocation serves as a simple marker for the maturational state of inner hair cells.

The transformation from spiking to non-spiking behavior in inner hair cells is driven by changes in ion channel properties, including the loss of calcium channels and the acquisition of new potassium channels, such as big-conductance calcium dependent potassium channels (BK) [17, 22]. The time course for BK channel acquisition is like that of nuclear translocation and endolymphatic potential in that it happens rapidly and at the onset of hearing (compare BK acquisition data from [17] to onset of endolymphatic potential shown here in Fig. 6). In contrast, other hair cell features, like cell size, increase much more slowly and happens progressively during the pre-hearing period [17].

### Developmental Gradients Across the Length of the Cochlea

We saw that nuclear migration occurred in all three cochlear turns, but this did not occur in synchrony. Nuclei began to move in the middle turn first, followed by the high-frequency-encoding basal turn and low-frequency-encoding apical turn. This wave is similar to the developmental waves seen at the very onset of hair cell development. In the mouse cochlea, progenitors in the basal turn differentiate into hair cells and supporting cells due to the expression of Atoh1 at embryonic day (E)13.5 [23]. A wave of differentiation continues towards the cochlear apex over the following few days [23, 24]. In a similar spatial gradient, transduction currents mature first in the mid-basal region at P0, and do not reach maturity in the apex until several days later at P2 [25]. If change in nuclear position is a marker for hair cell development, then, the maturational changes triggered at the onset of hearing also follow a developmental gradient which can be easily monitored by tracking gradients in nuclear position within the same epithelium.

### Nuclear Position is Stable in Post-natal Outer Hair Cells

Nuclear position did not change in the outer hair cells. The stability of nuclear position in outer hair cells stands in stark contrast to the mobility of inner hair cell nuclei. Indeed, as we show here, nuclear migration is part of the natural course of post-natal development for inner hair cell whereas keeping the nucleus at the base of the cell appears to be crucial for the normal function of outer hair cells [6, 26]. In mouse models with mutations to the proteins that connect the nucleo-plasma to the cyto-skeleton (**Li**nkers of **N**ucleoskeleton to **C**ytoskeleton, LINC complex), OHC nuclei float to the apex of outer hair-cells. These cells eventually die, resulting in deafness, although the reason for why the normal extreme basal position of nuclei in OHCs is significant is unclear. Curiously, the same LINC complex mutations that disrupt OHC nuclei appear to have only a modest influence on the position of inner hair cell nuclei [6], suggesting that the mechanisms controlling nuclear position may be different, even in two closely related cell types.

### What Triggers Nuclear Migration?

Several different mechanical, biophysical, and neuronal connectivity changes coincide with the onset of hearing. These changes include, amongst others, the opening of the ear canal for efficient transmission of sound input [27], loss of efferent innervation in inner hair cells [16, 28], the onset of endolymphatic potential to increase the driving force to hair cells [18], changes in endolymphatic calcium concentration [29], changes to the ion channel properties of the hair cell that take the cell from spiking to non-spiking [9], and changes to hormonal signals such as produced by fluctuations in thyroid hormones and receptors [30].

Although loss of efferent connections with inner hair cells is a characteristic change that occurs at the onset of hearing, this is unlikely to stimulate nuclear migration; nuclear position is normal both before and after the onset of hearing in a genetically modified mouse model with disruptions to efferent innervation (see Fig. 5 in [31]).

The rapid onset and temporal coincidence between ion channel expression and nuclear migration led us to hypothesize that a sudden increase in transduction currents produced by the dramatic rise in endolymphatic potential was triggering nuclear translocation and changes to ion channel composition. Our results show that immature inner hair cells do not automatically mature during a period in culture. Although tonic depolarization that partially mimics the influence of EP is required to maintain the mature pattern of BK channel expression in fully developed inner hair cells [13], culture conditions that impose a tonic depolarization on immature inner hair cells do not trigger nuclear migration or stimulate the emergence of BK channels. These results suggest that the mechanisms that trigger the maturation of inner hair cells are not simply linked to the tonic depolarization of hair cells induced by a positive EP.

### Postnatal Thyroid Hormone Expression May Play a Role in IHC Morphological Maturation

That thyroid hormone is critical to IHC development has been well-established [32]. In mice, thyroid hormone (TH) is expressed in IHCs between embryonic day (E)18 and P12 and is important for inner hair cells to mature in morphology and physiology. The hair cells in TH knockout animals fail to reach several maturational milestones. Inner hair cells develop normally until about P12 but remain immature well past the onset of hearing. The immature phenotype includes a failure or delay in acquiring BK channels [32, 33], persistence of efferent connections to inner hair cells and immature looking synapses [34, 35]. It is now also apparent that the nucleus itself remains positioned to the base of the cell at P14 whereas in the wild type mice, the nucleus has migrated away from the basal pole to the middle of the cell ([34] Fig. 2; [35] Fig. 7). The timeline for serum levels of thyroid hormone (T3) and its precursor (T4) is also important to consider when searching for maturational triggers in inner hair cells. In wildtype mice, T3 and T4 levels peak around the onset of hearing, between P12 and P16, and decrease by the third postnatal week [36, 37]. These results suggest that nuclear translocation and the ion channel maturation of inner hair cells may require natural hormonal signaling which were missing in our in vitro experiments. The data from the TH knockout mice suggest that nuclear migration and the biophysical maturation of inner hair cells are connected since disruptions to one coexist with dysfunction in the other. Whether the relationships are causal or are simply temporally coincident remain to be determined.

### Why Might Nuclear Position Matter?

Nuclear proximity to synapses may be favorable for synaptogenesis before the onset of hearing, perhaps by making it easier to deliver the components needed for forming synapses and for efficient recursive signaling between synapses and nucleus. Emerging research in skeletal muscle suggest that this might be the case for the successful formation of the neuromuscular junction (reviewed in [5]). There, the nuclei of multi-nucleated myocytes are conspicuously positioned close to the developing neuro-muscular junctions [38]. Mutations that disrupt this proximity often lead to synaptic malformations resulting in muscle pathologies [39, 40]. In the inner ear, the proximity of the nucleus to synapses in early post-natal days may similarly support a period of heightened synaptic plasticity. This idea is plausible given the dramatic refinement of afferent and efferent synapses on inner hair cells during the pre-hearing period (reviewed in [41]).

Mature function may rely on moving the nucleus away from synapses. In cone photoreceptors, nuclei are first positioned near forming synapses and then migrate away from the synaptic region towards the photosensitive outer segment [7]. In these cells, the efficiency of mature functioning synapses depends on the nucleus moving away from the synapse. When LINC complexes are disrupted and nuclear re-positioning does not occur [5], cone synapses have inefficient synaptic transmission and reduced dark sensitivity [8]. This suggests that while nucleo-synaptic proximity might be important for synaptogenesis, synaptic efficiency may require the nucleus to move away.

That the nucleus serves as an important marker for cellular sub-compartments has long been implicitly recognized in inner hair cells. Functionally distinct regions of inner hair cells are described by reference to the nucleus. For example, the synaptic region where dense-core ribbons and associated active zone proteins are found is often described as being confined to the “sub-nuclear” region. On the other hand, BK channels are confined to the supra-nuclear or “neck” region of inner hair cells [11]. This compartmentalization away from the synaptic region may be important for separating the synaptic and extra-synaptic regions to support the different roles of calcium in the mature cell [11]. Given that BK channels are placed with such precision to the supra-nuclear region, the position of the nucleus maybe an important cue for constraining the expression of ion channels to specific sub-cellular compartments.

Precise control of nuclear position is increasingly being recognized as important for the normal development and function of a wide range of cells [5]. Why nuclear position matters for these cells is not well understood but a prevalent hypothesis is that nuclear position plays a role in synaptogenesis, perhaps contributing to synaptic efficiency [8]. Here we described a previously unrecognized late-phase and rapid change in nuclear position in mammalian inner hair cells. The coincidence of the migration with the onset of hearing implicates nuclear position as being an important step for the final maturation of these cells. Whether this rapid onset of nuclear movement is responsive to or causally related to the functional transformation of inner hair cells and their synapses remains to be determined.

## Data Availability

The data supporting the findings of this study are either available within the article or by contacting the corresponding author.
